# Testing the Efficacy of Attitudinal Inoculation Videos to Enhance COVID-19 Vaccine Acceptance: Quasi-Experimental Intervention Trial

**DOI:** 10.2196/34615

**Published:** 2022-06-20

**Authors:** Rachael Piltch-Loeb, Max Su, Brian Hughes, Marcia Testa, Beth Goldberg, Kurt Braddock, Cynthia Miller-Idriss, Vanessa Maturo, Elena Savoia

**Affiliations:** 1 Department of Biostatistics Harvard TH Chan School of Public Health Boston, MA United States; 2 Emergency Preparedness Research Evaluation & Practice Program Division of Policy Translation & Leadership Development Harvard TH Chan School of Public Health Boston, MA United States; 3 Polarization and Extremism Research and Innovation Lab American University Washington, DC United States; 4 Massachusetts Association of Health Boards Wellesley, MA United States; 5 Jigsaw Google LLC New York, NY United States

**Keywords:** attitudinal inoculation, intervention, COVID-19 vaccine, vaccine hesitancy, COVID-19, vaccine, vaccination, public health, health intervention, misinformation, infodemiology, vaccine misinformation

## Abstract

**Background:**

Over the course of the COVID-19 pandemic, a variety of COVID-19-related misinformation has spread and been amplified online. The spread of misinformation can influence COVID-19 beliefs and protective actions, including vaccine hesitancy. Belief in vaccine misinformation is associated with lower vaccination rates and higher vaccine resistance. Attitudinal inoculation is a preventative approach to combating misinformation and disinformation, which leverages the power of narrative, rhetoric, values, and emotion.

**Objective:**

This study seeks to test inoculation messages in the form of short video messages to promote resistance against persuasion by COVID-19 vaccine misinformation.

**Methods:**

We designed a series of 30-second inoculation videos and conducted a quasi-experimental study to test the use of attitudinal inoculation in a population of individuals who were unvaccinated (N=1991). The 3 intervention videos were distinguished by their script design, with intervention video 1 focusing on narrative/rhetorical (“Narrative”) presentation of information, intervention video 2 focusing on delivering a fact-based information (“Fact”), and intervention video 3 using a hybrid design (“Hybrid”). Analysis of covariance (ANCOVA) models were used to compare the main effect of the intervention on the 3 outcome variables: ability to recognize misinformation tactics (“Recognize”), willingness to share misinformation (“Share”), and willingness to take the COVID-19 vaccine (“Willingness”).

**Results:**

There were significant effects across all 3 outcome variables comparing inoculation intervention groups to controls. For the Recognize outcome, the ability to recognize rhetorical strategies, there was a significant intervention group effect (P<.001). For the Share outcome, support for sharing the mis- and disinformation, the intervention group main effect was statistically significant (P=.02). For the Willingness outcome, there was a significant intervention group effect; intervention groups were more willing to get the COVID-19 vaccine compared to controls (P=.01).

**Conclusions:**

Across all intervention groups, inoculated individuals showed greater resistance to misinformation than their noninoculated counterparts. Relative to those who were not inoculated, inoculated participants showed significantly greater ability to recognize and identify rhetorical strategies used in misinformation, were less likely to share false information, and had greater willingness to get the COVID-19 vaccine. Attitudinal inoculation delivered through short video messages should be tested in public health messaging campaigns to counter mis- and disinformation.

## Introduction

The study of misinformation and disinformation and how to counter them is not new. There are centuries-old examples of the challenges in rebutting misleading or manipulative information [1-3]. However, although false and manipulative media are not new, “the digital age has changed how such messages are created, circulated, and interpreted, as well as their potential effects” [4]. As features of the COVID-19 media ecosystem, misinformation and disinformation are functionally similar, in that both either contradict or distort the current scientific and public health consensus as to the nature of the virus and appropriate steps to combat it [5,6]. However, the 2 terms refer to separate phenomena insofar as concerns motive. “Misinformation” is unintentionally inaccurate, while “disinformation,” is intentionally inaccurate and meant to mislead [7]. In the context of public health, the term “infodemic” was coined to refer to “an overflow of information of varying quality that surges across digital and physical environments during an acute public health event” [8]. Infodemiology, as a field of study and intervention, dates back to 1996 [9,10]. Eysenbach defines infodemiology as the “science of distribution and determinants of information in an electronic medium, specifically the Internet, or in a population, with the ultimate aim to inform public health and public policy” [9]. As Eysenbach describes it, infodemiology rests on the premise that public health and patterns of communication are correlated, and perhaps even causally connected.

Since the pandemic’s beginning, a variety of COVID-19-related misinformation and disinformation has spread and been amplified online [11]. The content and spread of misinformation can influence COVID-19 beliefs and protective actions [12,13]. Despite the availability of the COVID-19 vaccine in the United States, hesitancy among the general population remains a challenge. In their review of 39 nationally representative polls taken in the first half of 2021, Steelfisher et al [14] found that nearly 30% of the population remains hesitant to get the COVID-19 vaccine. Belief in vaccine misinformation is associated with lower vaccination rates and higher vaccine resistance [15]. The spread of misinformation and disinformation online can increase COVID-19 vaccine hesitancy [16]. Studies conducted at varying time points in 2020 have found that reliance on social media is associated with higher levels of holding both conspiracy beliefs and higher levels of vaccine hesitancy [17-20].

Studies of how to address the current infodemic are nascent. The inaugural World Health Organization (WHO) Infodemiology Conference of 2021 called for more research on interventions to address the infodemic [11]. Countering misinformation is a critical piece of infodemic management because misinformation impacts protective actions and vaccine hesitancy. Infodemiology research has shown that quality health information can be elusive to the public, especially in evolving situations, such as a pandemic [21]. One common approach used by public health risk communicators focuses on “facts.” However, as Eysenbach [21] points out, in times of evolving science, factual information can be hard to determine, and initial reports and decisions are made based on the best information available at any given time. Currently, the most common approach to countering misinformation is to engage in fact checking. Research evaluating the utility of online fact checking suggests that even under less uncertain conditions, it remains an uneven but relatively effective counterstrategy to disinformation [22-26]. However, fact checking carries with it 2 challenges: asymmetry and volume. Feelings of social ostracism are shown to decrease receptivity to counter disinformation fact checking [27]. Media consumers with less overall political knowledge are likewise less receptive, as are political conservatives more generally [28]. Meanwhile, the sheer volume with which bad actors are increasingly equipped to “flood the zone” with mis- and disinformation [29] can exhaust most audience’s ability to sift good information from bad, apart from more formal, time-and-resource-intensive fact-checking projects. Human moderators cannot match the speed and volume of false information and, furthermore, require an ever-changing range of subject expertise that content moderators cannot reasonably be expected to acquire [30,31]. Studies into the relative efficacy of logic-based versus emotionally based public health communication have suggested that the use of narrative [32-34], appeals to values [35,36], and rhetoric of personal, lived experience [37-39] yield better persuasive outcomes than more abstract, fact-based, or logical counterparts. Per Maertens et al [40], this might relate to the “broad spectrum” of potential viewpoints that such approaches address. That is, fact checking’s narrower focus on specific *content* addresses fewer points of persuasive vulnerability than a broader focus on *form* offered by rhetoric, narrative, and values.

Attitudinal inoculation (or, simply, “inoculation”) is a preventative approach to combating misinformation and disinformation that leverages the power of narrative, rhetoric, values, and emotion. Inoculation theory promises that people can become resistant to persuasion if they perceive a threat from an attempt to change their beliefs or attitudes and if they receive information to refute this attempt [41]. It originates in the midcentury work of William McGuire [41-43]. It uses the biological metaphor of viral inoculation to propose that “[t]hrough exposing individuals to messages containing a weakened argument against an attitude they hold, it is possible to ‘inoculate’ the individuals against future attacks on the attitude” [44]. Inoculation consists of exposing someone to a persuasive message that contains weakened arguments against an established attitude, which develops resistance against stronger persuasive attacks in the future [41].

Inoculation is preemptive, addressing audiences holding “healthy (ie, preferred) positions,” or agnostic and undecided [45]. It scales against the “flooded zone” of information, allowing individuals to bypass entire categories of misleading, manipulative, or simply distracting information. Inoculation is suited to address the needs of low-information audiences, ideologically polarized and conspiratorial groups, and groups that are traditionally difficult audiences to reach with corrections [46]. Inoculation may partially overcome the post hoc correction challenges of asymmetry and volume, while accounting for variations in the efficacy of fact-based versus narrative/rhetoric-based approaches.

Studies have supported the effectiveness of attitudinal inoculation as a tool for strengthening resistance to persuasion on public health topics, such as underage alcohol consumption, adolescent smoking initiation, deceptive nutrition-related food claims, unprotected sex, and child vaccine safety claims [47,48]. Additionally, attitudinal inoculation has been shown as an effective strategy for counterradicalization. In a foundational study, inoculation conferred resistance to persuasion by far-right and far-left extremist propaganda by reducing the credibility of the extremist groups that produced the propaganda and increasing reactance (the combination of anger and counterarguing) against the propaganda itself. By reducing source credibility and increasing reactance, inoculation ultimately reduced participant intentions to support the group that produced the propaganda [49].

The potential for attitudinal inoculation to combat COVID-19 vaccine misinformation was proposed by van der Linden et al [50]. Although attitudinal inoculation enjoys a rich body of literature, and infodemiology likewise can claim extensive source material, the specific application of both approaches to the crisis of the COVID-19 pandemic is scant at best. This study is among the first to answer the call made by van der Linden et al [50]. It not only sought to test the effectiveness of attitudinal inoculation against COVID-19 misinformation and disinformation but also attempted to address questions relating to persuasive communication, which bear direct relevance to the matter of public health communication in the pandemic. As described before, the relative efficacy of fact versus narrative or rhetoric in persuasive messaging has been studied across many dimensions of public health. Our study testing the use of video-based attitudinal inoculation to inoculate viewers against misinformation on COVID-19 vaccine injury is the first of its kind to compare the effectiveness of using facts versus narrative-rhetoric approaches to attitudinal inoculation messages relating to COVID-19 vaccine misinformation and disinformation. The goal of our research was to build upon the work of Braddock, van der Linden, and other inoculation theorists by using inoculation messages in the form of short video messages to promote resistance against persuasion by COVID-19 vaccine misinformation.

## Methods

### Identification of Antivax Narratives

This study was built upon our formative evaluation work that identified common rhetorical strategies and COVID-19 vaccine misinformation narratives and used formative surveys to explore their prevalence and validate select survey items that were used in this study [20,51,52]. The narratives were identified by analyzing 6 months of content from 10 online channels of antivaccine or COVID-19 denialist propaganda. These took the form of Twitter accounts, amateur videos, documentaries, Facebook groups, blogs, and Instagram pages. From these media sources, we created a list of 22 key narrative tropes and 16 rhetorical strategies, which represented the discursive foundation of the antivaccine and COVID-19 denialist media data collected, and created a codebook [53]. Narratives ranged from general claims that the COVID-19 vaccine could cause physical injury to the theory it was a bioweapon promoted by intelligence agencies for shadowy and perhaps even supernatural purposes. Some rhetorics framed their arguments along the lines of bodily autonomy by co-opting the language of women’s reproductive rights, while others relied on audio-visual cues, such as nauseating colors and low-frequency sounds, to cue unease in their audience.

### Development of Inoculation Messages

Based on the identification of the antivax narratives, we selected a prominent metanarrative related to vaccine injury that was used to develop 3 different inoculation messages: (1) a fact-based video, focused on countering false statistics about the science and safety of vaccines; (2) a narrative and rhetoric-focused video, which “prebunked” (ie, practice of countering potential misinformation by warning people against it before it is disseminated) common antivaccine misinformation strategies; and (3) a hybrid video that tested a combination of factual rebuttal with narrative/rhetorical prebunking. These 3 approaches were selected in order to deepen understanding of the relative efficacy of fact-based and narrative/rhetoric-based persuasion. As discussed before, the relative efficacy of each approach has been addressed across a variety of fields, from extremist deradicalization to public health.

### Development of Inoculation Videos

We designed a series of 30-second inoculation videos and conducted a quasi-experimental study to test the use of attitudinal inoculation in a population of individuals who were unvaccinated. We developed 3 inoculation videos. Each 30-second video contained a “microdose,” a weakened example of manipulation, which has been shown not to cause harm in a controlled research setting. The microdoses, while weakened, constitute an “active threat” that let people generate cognitive “antibodies” [40,54,55]. The differences between the 3 inoculation videos are shown in Table 1.

**Table 1:** Description of video types.

Video types	Purpose	Example of video script
Narrative and rhetoric inoculation	Inoculate viewers against vaccine misinformation strategies, such as manipulation, scapegoating, or conspiratorial reasoning.	“Sometimes, people trying to change your mind this way will show pictures of needles, crying babies, or extreme close-ups of viruses. Sometimes, they’ll make videos with sounds that are scientifically proven to provoke a feeling of unease in humans. Strange, but true!”
Factual rebuttal inoculation	Counter false information about science and safety about vaccines.	“Sometimes, these people talk about ‘vaccine injury’. Actual injuries related to vaccines are extremely rare. Only two out of every one million people who received vaccine results even claimed to have been injured. Of those claims, about a third turned out not to be actual injuries related to vaccines.”
Hybrid	Combine fact-based information and inoculation against misinformation strategies.	“Sometimes, these people talk about ‘vaccine injury’. Actual injuries related to vaccines are so rare, you are nearly twice as likely to be struck by lightning.” “Actual injuries related to vaccines are extremely rare. You are 769 times more likely to die from COVID than to experience any vaccine injury.” “Sometimes, people trying to change your mind this way will show pictures of needles, crying babies, or extreme close-ups of viruses. Sometimes, they’ll make videos with sounds that are scientifically proven to provoke a feeling of unease in humans. Strange, but true!”

### Study Hypothesis

Based upon our prior research on the relationship between knowledge, attitude, and behavior, we posited 3 hypotheses:

- Hypothesis 1: Relative to noninoculated participants, inoculated participants will demonstrate a greater ability to identify rhetorical strategies typically used in mis- and disinformation videos.
- Hypothesis 2: Relative to noninoculated participants, inoculated participants will be less likely to report engaging in behaviors that support the spread of COVID-19 mis- and disinformation videos.
- Hypothesis 3: Relative to noninoculated participants, inoculated participants will report greater intention to get vaccinated against COVID-19.

### Study Design

**Figure 1:**
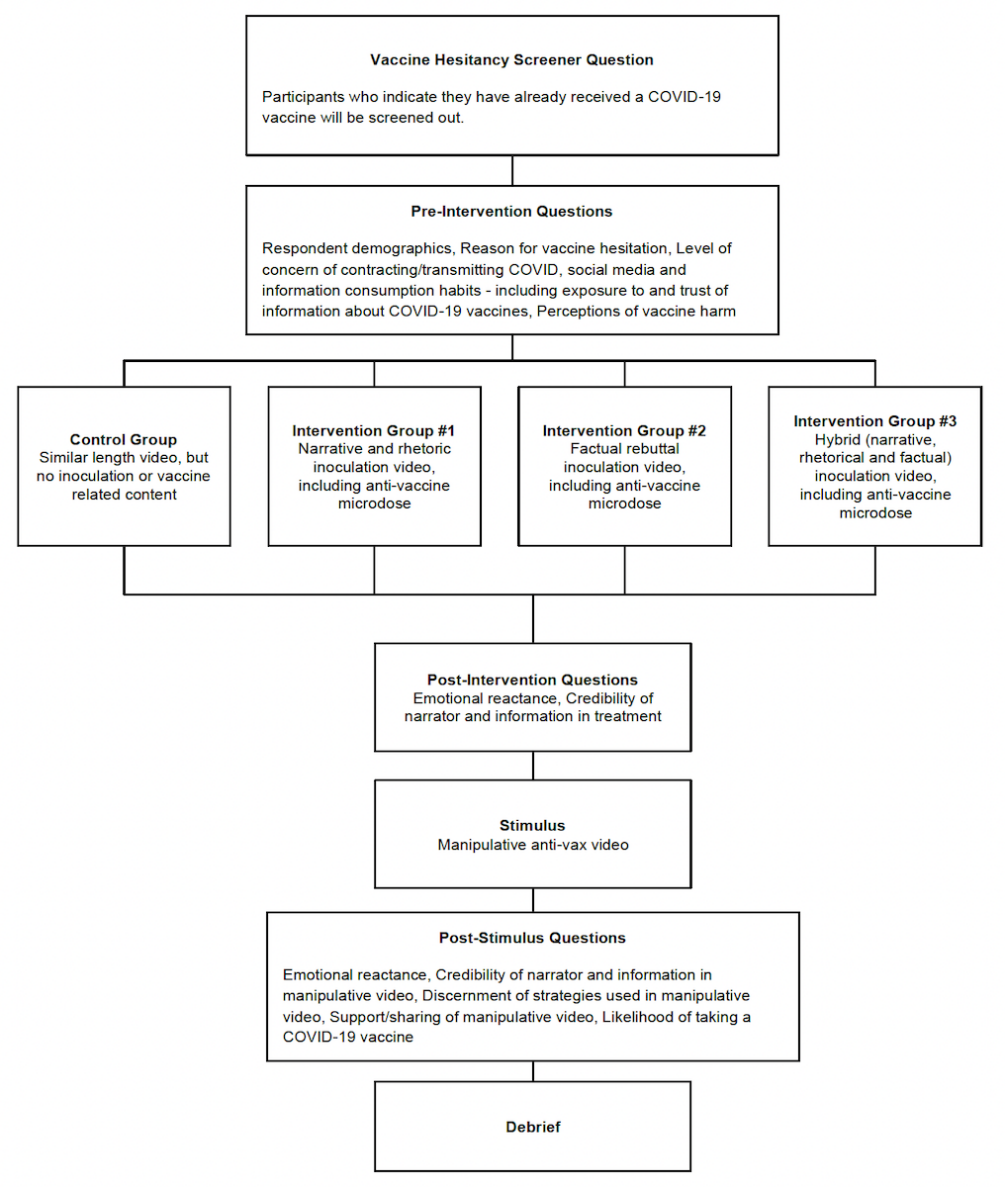
Study design.

We conducted a quasi-experimental study with a pre-post intervention questionnaire and control group using an organic sampling survey method between June 3 and 5, 2021 [56]. Using this method, 4 separate surveys were conducted for each of the video exposure interventions, and as such, randomization was not possible. We conducted our surveys through the Pollfish (an online survey company) survey platform. Participants were eligible to participate if they indicated they had *not* received a COVID-19 vaccine, were over the age of 18 years, and lived in the United States. In total, 500 US adults were recruited into each study arm by Pollfish via mobile technology. Respondents were recruited into 1 of 4 study arms, 1 for each type of inoculation message, plus a control group that received a video unrelated to inoculation or vaccines using the design outlined Figure 1.

All participants first answered questions about their demographics, social media and information consumption, exposure to and trust of information about COVID-19 vaccines, and perceptions of vaccine harm. Then, participants in the 3 treatment groups were “inoculated” by showing them a 30-second scripted video that highlights narrative or rhetorical tactics used in vaccine misinformation (intervention group 1, “Narrative,” n=500), contains factual rebuttal of vaccine misinformation (intervention group 2, “Fact,” n=500), or a hybrid of both (intervention group 3, “Hybrid,” n=500). The control group (n=500) watched a neutral video that described how to make a paper airplane. Participants were then asked a series of questions on their perceptions of the video.

After watching the inoculation video, participants were shown a video stimulus that utilizes the manipulation techniques participants were alerted to in the inoculation video. The same stimulus video was displayed to participants in the control group and all treatment groups. Participants were then asked the same series of questions on their perceptions of the video. The same questions were asked following the intervention/control video and the stimulus video in order to avoid alerting the respondent as to the type of video being assessed.

### Ethical Considerations

The study protocol was approved by the American University Institutional Review Board (IRB-2022-295).

### Variables of Interest

We had 3 hypotheses of interest. Each hypothesis corresponded to a different dependent variable below. The first hypothesis was related to the ability to identify misinformation, the second was related to willingness to share information, and the third was related to willingness to get vaccinated. The measures described below were designed to correspond to these 3 research questions, though the survey also included other items related to emotional reactance to the content.

#### Dependent Variables (Question Number - Variable Name)

We selected 3 particular outcomes of interest:

- The ability to recognize rhetorical strategies in a video containing misinformation about the COVID-19 vaccine, such as unusual colors, scary music, and vague language (Q25 - Recognize)
- Willingness to share the video containing misinformation about the COVID-19 vaccine (Q24 - Share)
- Willingness to get vaccinated (Q26 - Willingness)

#### Independent Variables

We had the following independent variables:

- Demographic variables: gender (male, female), age (continuous), race (non-Hispanic White, non-Hispanic Black, Hispanic, non-Hispanic Asian, other), income (continuous), education (less than high school, high school/General Educational Development (GED), some college, bachelor’s degree, postgraduate degree, other)
- General vaccine attitude covariates: “Most vaccines do not cause immediate injuries or side effects,” “Most vaccines do not lead to long-term side effects,” “Vaccines cause more harm than benefit,” “Taking a vaccine is likely to give me a disease,” “Vaccination can protect me from getting a disease,” “Vaccines cause autism,” and “Vaccines are designed as a form of government control,” which was tested and then analyzed as a factor that controlled for vaccine attitudes (see analysis plan)

### Statistical Analyses

The analyses included 3 steps: first, to conduct descriptive statistics of baseline sample characteristics; second, to analyze multi-item scale development; and third, to test the effect of the 3 interventions in comparison to controls on the endpoints collected after the second video. All final models were adjusted for demographics (age, gender, race, education, and income); baseline value, if available; and mean scores calculated about general attitudes toward vaccines.

#### Descriptive Statistics of Baseline Sample Characteristics

Baseline characteristics of the sample, including age, gender, race, income, education, and attitudinal variables, were described using means and SDs for continuous variables and counts and percentages for categorical variables. Descriptive statistics were calculated for the entire sample and by intervention group.

#### Multi-Item Scale Development

Principal component analysis (PCA) was used to test the dimensionality of multi-item scales, which included the scales for prior general vaccine attitudes and the 3 primary study outcomes, namely Recognize, Share, and Willingness. We planned to retain components associated with eigenvalues greater than 1 as long as factor loadings and internal consistency within components, as measured by Cronbach α, were acceptable (>.7). When more than 1 component was retained, the varimax rotation was applied to the model to aid in the interpretation of the factor loadings. For retained components, scores were created as the mean of their items.

#### Statistical Analysis of Effects of Interventions on Outcomes

Analysis of covariance (ANCOVA) models were used to compare the main effect of the intervention on the 3 outcome variables. The model for each outcome included the main effect of the intervention adjusted for age, gender, race, education, income, and the scores for prior general attitudes toward vaccines that was formed in the second step of our analysis. For the Share scale, the model also included the baseline Share score from after the first video. For the 3 outcomes, we were interested in testing the null hypothesis of no difference between intervention groups using a 2-tailed statistical test. To control the type 1 error rate resulting from multiple endpoints, we used the Holm method. For outcomes with a significant intervention group *F* test, we tested paired differences between the intervention groups and the control group and controlled for multiple comparisons using the Dunnett test. The power of the statistical test for an ANOVA with 3 intervention groups and 1 control group where the Dunnett test is used to compare each treatment mean with the control mean, one would require 477 subjects in each group in order to achieve 81% power to detect a mean difference of 0.33 between at least 1 pair of intervention and control groups, assuming an SD of 1.8 within each group and a family-wise type 1 error rate of .017, which corresponds to the first rejection level of the Holm method for this study.

## Results

### Sample Characteristics

This analysis included 1991 subjects. Although 500 (25.1%) subjects were enrolled in each intervention group, not all subjects completed the survey questionnaire, leaving an analysis population of 495 (24.9%) participants in the control group and 480 (24.1%) in the narrative-rhetorical, 489 (24.6%) in the factual, and 489 (24.6%) in the hybrid video intervention groups. Overall, the study population had a mean age of 40.7 (SD 11.8) years, and 968 (50%) were female, 1439 (74%) were non-Hispanic White, 1173 (60%) had a bachelor’s degree or higher education, and 773 (40%) reported an income of US $100,000 or more. A summary of baseline characteristics for the entire sample and by intervention group is given in Table 2.

**Table 2:** Baseline characteristics.

Characteristic		All groups (N=1953)	Controls (N=495)	Narrative (N=495)	Fact (N=489)	Hybrid (N=489)	*P* value^a^	
Age (in years), mean (SD)		40.8 (11.8)	41.0 (11.7)	36.6 (11.6)	41.6 (11.4)	43.6 (11.4)	<.001	
Female gender, n (%)		968 (50)	248 (50)	234 (49)	243 (50)	243 (50)	.98	
**Race, n (%)**								.03
	Non-Hispanic White	1439 (74)	380 (77)	347 (72)	356 (73)	356 (73)	N/A^b^	
	Non-Hispanic Black	206 (11)	29 (6)	59 (12)	59 (12)	59 (12)	N/A	
	Hispanic/Latino	98 (5)	35 (7)	21 (4)	21 (4)	21 (4)	N/A	
	Non-Hispanic Asian	73 (4)	13 (3)	20 (4)	20 (4)	20 (4)	N/A	
	Other	137 (7)	38 (8)	33 (7)	33 (7)	33 (7)	N/A	
**Income (US $), n (%)**								.02
	<25,000	238 (12)	76 (15)	54 (11)	54 (11)	54 (11)	N/A	
	25,000-49,999	245 (12)	75 (15)	56 (11)	57 (12)	57 (11)	N/A	
	50,000-74,999	273 (14)	48 (10)	75 (15)	75 (15)	75 (15)	N/A	
	75,000-99,999	310 (16)	74 (15)	78 (16)	79 (16)	79 (16)	N/A	
	100,000-124,999	170 (9)	42 (8)	42 (9)	43 (9)	43 (9)	N/A	
	125,000-149,999	200 (10)	51 (10)	47 (10)	51 (10)	51 (10)	N/A	
	≥150,000	403 (21)	83 (17)	106 (22)	107 (22)	107 (22)	N/A	
	Prefer not to say	114 (6)	46 (9)	22 (5)	23 (5)	23 (5)	N/A	
**Education, n (%)**								<.001
	Less than high school	164 (8)	17 (3)	49 (10)	49 (10)	49 (10)	N/A	
	High school/GED^c^	290 (15)	103 (21)	61 (13)	63 (13)	63 (13)	N/A	
	Some college	254 (13)	104 (21)	50 (10)	50 (10)	50 (10)	N/A	
	Bachelor’s degree	405 (21)	105 (21)	100 (21)	100 (20)	100 (20)	N/A	
	Postgraduate degree	768 (39)	166 (34)	196 (41)	203 (42)	203 (42)	N/A	
	Other	72 (4)	0 (0)	24 (5)	24 (5)	24 (5)	N/A	

^a^Test of significant intervention group effect using ANOVA model for Age and Pearson chi-square test for categorical variables.

^b^N/A: not applicable.

^c^GED: General Educational Development.

### Results of Statistical Analysis of Scales

PCA of the 7 items measuring prior attitudes toward vaccines retained 2 factors that accounted for 65% of the variance in the data. All items had a high factor loading on 1 of the 2 factors, with loading in the range of 0.71-0.83, while also having small factor loadings of less than 0.2 on the other factor (Table 3). Items loading most heavily on the first component asked the level of agreement to the statements “Most vaccines do not cause immediate injuries or side effects,” “Most vaccines do not lead to long-term side effects,” and “Vaccination can protect me from getting a disease,” while the remaining items that asked “Vaccines cause more harm than benefit,” “Taking a vaccine is likely to give me a disease,” “Vaccines cause autism,” and “Vaccines are designed as a form of government control” loaded highly onto the second component. Cronbach α for the 3 items loading on the first component was .70, while the 4 items loading on the second component had a Cronbach α of .83, indicating good internal consistency within each set of items. Because these 7 items formed 2 distinct constructs with good reliability, we created 2 scores for general attitudes toward vaccines by taking a subject’s mean response to the questions that loaded highly onto each factor.

For the 3 questions measuring the Recognize outcome, PCA showed the items to be unidimensional, with a single factor accounting for 72% of the variance. Factor loadings were all greater than 0.84, and Cronbach α was .81. PCA of the 5 items measuring the Share outcome also retained a single factor that accounted for 70% of the variance in the data. Factor loadings were high (0.70-0.89), and Cronbach α was .89.

**Table 3:** PCA^a^ factor structure.

Construct items			Factor 1 loading		Factor 2 loading
**General attitudes toward vaccines**					
	Most vaccines do not cause immediate injuries or side effects.	0.79		0.13	
	Most vaccines do not lead to long-term side effects.	0.69		0.11	
	Vaccines cause more harm than benefit.	0.09		0.74	
	Taking a vaccine is likely to give me a disease.	0.09		0.75	
	Vaccination can protect me from getting a disease.	0.49		0.10	
	Vaccines cause autism.	0.19		0.75	
	Vaccines are designed as a form of government control.	0.14		0.66	
**Recognize**					
	Scary music	0.78		N/A^b^	
	Weird colors	0.78		N/A	
	Vague language (words that are unclear or not specific)	0.73		N/A	
**Sharing**					
	How likely are you to share this second video with people in your social media network?	0.83		N/A	
	How likely are your friends to share this second video on their social media networks?	0.81		N/A	
	If you could, how likely would you be to support the producer of this second video by following them (receiving future posts from them) on social media?	0.88		N/A	
	If you could, how likely would you be to financially support the producer of this second video?	0.82		N/A	
	How likely are you to check the facts on the second video you just watched?	0.60		N/A	

^a^PCA: principal component analysis.

^b^N/A: not applicable.

### Effect of Interventions on Outcomes of Interest

#### Hypothesis 1. The Ability to Recognize Rhetorical Strategies in a Video Containing Misinformation About the COVID-19 Vaccine (Recognize)

For the Recognize score, the ability to recognize rhetorical strategies, there was a significant intervention group effect (*F*_(3,1929)_=8.5, *P*<.001); see Table 4. Since this was the smallest of the intervention group effect *P* values among the 3 study endpoints, the Holm method rejected the null hypothesis of the no-intervention-group effect when *P*<.05/3=.02, which was achieved. The least squares (LS) means (SE) of the Recognize scale for controls and the 3 video intervention groups were 3.67 (0.09), 3.98 (0.09), 4.10 (0.09), and 4.14 (0.09), respectively. The LS mean differences between intervention groups (Narrative, Fact, and Hybrid) and controls were 0.31 (*P*=.01), 0.43 (*P*<.001), and 0.47 (*P*<0.001), respectively, with all *P* values being significant after adjusting for multiple comparison using the Dunnett test. These tests indicated that each intervention group had a statistically significant greater awareness of the tactics used to gain attention in the second video compared to controls.

**Table 4:** ANCOVA^a^ models’ estimated intervention effects, mean scores, and differences.

Intervention LS^b^ means and SEs					Differences from control intervention	
Outcome by intervention		*F* statistic *P* value^c^	LS mean (SE)^c^	LS mean (SE)		Adjusted *P* value^d^
**Recognition of rhetorical strategies (“Recognize”)**						
	Intervention effect	<.001	N/A^e^	N/A		N/A
	Control	N/A	3.67 (0.09)	N/A		N/A
	Narrative	N/A	3.98 (0.09)	0.31 (0.11)		.01
	Fact	N/A	4.10 (0.09)	0.43 (0.10)		<.001
	Hybrid	N/A	4.14 (0.09)	0.47 (0.10)		<.001
**Willingness to share misinformation content (“Share”)**						
	Intervention effect	.017	N/A	N/A		N/A
	Control	N/A	4.11 (0.08)	N/A		N/A
	Narrative	N/A	3.90 (0.07)	–0.21(0.09)		.03
	Fact	N/A	3.90 (0.07)	–0.22 (0.09)		.022
	Hybrid	N/A	3.89 (0.07)	–0.22 (0.09)		.019
**Willingness to get vaccinated (“Willingness”)**						
	Intervention effect	.006	N/A	N/A		N/A
	Control	N/A	2.77 (0.09)	N/A		N/A
	Narrative	N/A	3.05 (0.09)	0.28 (0.10)		.012
	Fact	N/A	3.05 (0.09)	0.28 (0.10)		.011
	Hybrid	N/A	3.05 (0.09)	0.28 (0.10)		.01

^a^ANCOVA: analysis of covariance.

^b^LS: least squares.

^c^*F* statistic, LS mean, and SE were obtained from an ANCOVA model for each outcome, with the intervention group as the main effect and adjusting for age, gender, race, education, income, and scores from 2 scales of general attitudes toward vaccines.

^d^*P* value adjusted for multiple comparisons between controls and intervention groups using the Dunnett test after finding a significant main effect for intervention.

^e^N/A: not applicable.

#### Hypothesis 2. Support for Sharing the Video Containing Misinformation About the COVID-19 Vaccine (Sharing)

For the Share scale, support for sharing the mis- and disinformation, the intervention group main effect was statistically significant (*F*_(3,1928)_=3.4, *P*=.02) using the Holm *P* value threshold of .05. For the control and intervention groups (Narrative, Fact, and Hybrid), the LS means (SE) were 4.11 (0.08), 3.90 (0.07), 3.90 (0.07), and 3.89 (0.07), respectively. The LS means for the difference between the control group and the 3 intervention groups were 0.21 (*P*=.03), 0.22 (*P*=.022), and 0.22 (*P*=.019) lower than that for the control group, respectively, indicating lower support for sharing/supporting the second video in each intervention group compared to controls.

#### Hypothesis 3. Willingness to Get vaccinated (Willingness)

The intervention group effect for the Willingness scale, willingness to get the vaccine, had the second smallest *P* value (*F*_(3,1929)_=4.1, *P*=.01) among the 3 study endpoints, which was significant at the Holm method adjusted cut-off of 0.05/2=.03. The LS means (SE) for the willingness scale for the control and 3 intervention groups were 2.77 (0.09), 3.05 (0.09), 3.05 (0.09), and 3.05 (0.09), respectively. The difference between the control groups and the 3 intervention groups (Narrative, Fact, and Hybrid) were 0.28 (*P*=.012), 0.28 (*P*=.011), and 0.28 (*P*=.01) points higher than that for the controls, respectively, indicating that subjects in the intervention groups were more willing to get the COVID-19 vaccine compared to controls.

## Discussion

### Principal Findings

The purpose of this study was to identify the effect of attitudinal inoculation videos on the ability to identify misinformation, willingness to share misinformation, and willingness to vaccinate. We found there was a significant effect of having been exposed to an inoculation video compared to a control video across each outcome of interest. Our results support our initial hypotheses that inoculation messaging can increase the ability to identify misinformation, decrease willingness to share misinformation, and increase willingness to vaccinate.

Our study explored not only whether there is an effect of inoculating participants but also whether inoculating against a narrative/rhetorical strategy or against factual misinformation is more effective. We also tested a hybrid video that combined fact-based and narrative/rhetorical strategies within the inoculation message. Past studies into the relative efficacy of narrative/rhetoric compared to fact-based appeals have suggested that narrative approaches are more likely to be persuasive to viewers [32-34,37-39,55]. However, we did not find significant differences across the 3 active intervention groups, suggesting that the content of the intervention video (narrative/rhetoric, fact based, or hybrid) does not impact the effect of the intervention. This may be attributable to the nuanced differences in the scripts used, which might have been difficult to distinguish in such a short time frame. Additionally, because there was only 1 video for each video script strategy, it is not possible to say whether there may be differences in other videos that used these same strategies. It is even possible that the cinematographic and casting choices behind each video (ie, the production and the medical workers who narrated them) themselves made indirect narrative and rhetorical appeals to viewers. These appeals include, for example, clean sets, approachable body language, hopeful music, and other emotionally engaging features of the videos. This, in turn, might have produced a “flattening” effect, rendering the appeals of the videos more uniform.

The intervention videos were designed to protect people against being misled by flawed argumentation used in common online mis- and disinformation, such as conspiracy theories [57,58]. In practice, this means that watching a video with an inoculation message and a weakened microdose of manipulation techniques allows viewers to discern more readily subsequent misinformation that makes use of similar flawed argumentation techniques. In this study, we were able to achieve this in videos that were only 30 seconds in duration. This is critical because 30 seconds is consistent with short attention spans for online video consumption on social media and the length of many ads online, enabling such a video to be shown in ad slots. Prior inoculation studies have tested longer-form videos or text [49,51,58]. These findings for the effects of 30-second videos have implications for the ability to disseminate inoculation messages in social media ad slots.

Furthermore, online platforms are a viable place to disseminate these interventions to affect perceptions and behavior, because information on vaccine injury is viewed and spread on social media and exposure to online misinformation is associated with lower vaccination intentions [17,59,60]. Social media platforms that may hesitate at the prospect of hosting inoculation videos containing a microdose of misinformation might be reassured that in the proper context of an inoculation message, these microdoses are vital to the overall discrediting of misinformation and disinformation.

### Limitations

This study has limitations. First, the study sample was not representative of the general population but rather was more highly educated, mobile app users. Testing the effects of the intervention with a broader audience and determining whether there are differential effects among particular subsamples of the population will add to the understanding of the effect of these types of videos. Second, as described before, further investigation is needed to explore whether there may be meaningful differences in narrative versus fact versus hybrid models that could not be detected in this study due to the study design; however, in general, our results suggest that fact-based rhetorical strategies can be as effective as a narrative-rhetoric or hybrid approach.

### Conclusion

As an infodemic management strategy, approaches that go beyond fact checking, and do not simply focus on countering 1 piece of content, may be valuable. We found that attitudinal inoculation in video-based messages may be an intervention strategy that can be used in designing public health messaging campaigns to counter mis- and disinformation. Online dissemination of these videos could be a viable strategy to increase vaccine uptake and can be tried more broadly. Videos that use attitudinal inoculation to combat COVID-19 vaccine misinformation should be tested with a broader audience beyond the United States and on social media platforms, such as YouTube and TikTok. More research is needed to understand how videos with attitudinal inoculation perform when individuals are in a typical information consumption environment and faced with competing demands for attention.

## Abbreviations

ANCOVA: analysis of covariance
GED: General Educational Development
LS: least squares
PCA: principal component analysis
